# Effect of Piroxicam on ART Outcome: A Pilot Study

**Published:** 2014-11-01

**Authors:** Farnaz Sohrabvand, Fedyeh Haghollahi, Masoomeh Maasomi, Mamak Shariat

**Affiliations:** 1 Department of Infertility, Vali-e-Asr Hospital , Imam Khomeini Hospital Complex, Tehran University of Medical Sciences, Tehran, Iran; 2 Vali-e-Asr Reproductive Health Research Center, Vali-e-Asr Hospital , Tehran University of Medical Sciences, Tehran, Iran; 3 Maternal, Fetal and Neonatal Research Center, Tehran University of Medical Sciences, Tehran, Iran

**Keywords:** Embryo Transfer, Piroxicam, Pregnancy Rate, Assisted Reproductive Techniques

## Abstract

**Background:**

One of the most important factors affecting success rates in assisted reproductive techniques (ART) besides the number of oocytes retrieved and high quality embryos derived from them is the technical aspects of embryo transfer. It seems that pretreatement with uterine relaxants can be helpful in preventing un- pleasant cramps which can have an adverse effect on ART outcome. In this respect, some drugs such as prostaglandin inhibitors or sedatives have been evaluated but not confirmed yet remain controversial. This study was performed in order to assess the effect of administrating Piroxicam prior to embryo transfer on pregnancy rates in ART cycles.

**Materials and Methods:**

This pilot study was performed from August 2010 through December 2011 on 50 infertile women in ART cycles. Recombinant follicle stimulating hormone (rFSH) with a long gonadotropin releasing hormone (GnRH) analogue protocol were used for controlled ovarian hyperstimulation. The subjects were randomly allocated into two groups of 25 patients after obtaining written consent. Group A received a 10 mg Piroxicam capsule 30 minutes before embryo transfer and group B was the control group with no treatment. Data were analyzed by Chi-square and analysis of variance (ANOVA).

**Results:**

Pregnancy rate was 34% (n=17) totally, with 32% (n=8) in group A and 36% (n=9) in group B (p=0.75). Uterine cramps were experienced by 4 women (16%) in group B, while none were reported by women in group A (p=0.037).

**Conclusion:**

It seems that Piroxicam administration 30 minutes prior to embryo transfer can- not increase pregnancy rates, but can prevent or reduce uterine cramps after the procedure.

## Introduction

Assisted reproductive techniques (ART) have contributed tremendously to the infertility treatment. As experience has accumulated in the past three decades, success rates have increased, making them applied worldwide (1). Different factors contribute to the success rates in the various stages of these procedures. One of the most important factors affecting success rates besides the number of oocytes retrieved, high quality embryos and uterine receptivity is the technical aspects of embryo transfer (2). Due to the vast research performed in recent years, the embryo transfer technique has improved in different aspects including the type of catheters used and transfer of embryo under sonographic guidance. Also the effect of different factors such as administration of antibiotics and bed rest after transfer have been evaluated (3, 4). Regarding uterine factors, the absence of uterine contractions at the time of embryo transfer is reported to significantly affect endometrial receptivity (5, 6). Embryo transfer is an aggressive procedure that may induce endometrial inflammatory reaction and augmented myometrial contractility. In a non-pregnant uterus, uterine contraction patterns play an important role in human reproduction. It has been shown in different studies that suitable stimulation or prevention of uterine contractions after embryo transfer can increase the fertility rate (5, 7, 8).

In order to reduce uterine cramps, it is highly recommended to perform embryo transfer with the least trauma. In a study by Fanchin, they showed an increase in random fundocervical uterine contractions in cases of difficult embryo transfer (9). In another study, it was shown that applying a tenaculum to the cervix during a mock embryo transfer (ET) can increase uterine contractions (10).

Uterine contractions are induced by prostaglandin (PG) which is synthesized from arachidonic acid by cyclo-oxygenase (COX). It seems that pretreatement with uterine relaxants can be helpful in preventing unpleasant cramps. Theoretically non-steroidal anti-inflamatory drugs (NSAIDs) which block the action of COX can inhibit the production of PG and should have beneficial effect on pregnancy rates (11). In this respect some drugs such as prostaglandin inhibitors or sedatives have been evaluated but not confirmed, yet remain controversial. In a study by Bernabeu, in egg donation cycles, implantation rates did not show significant difference in oocyte recipients who had received indomethacin before transfer (12). In our previous study we compared the effect of indomethacin to hyoscine given before embryo transfer on ART outcome. It was shown that hyoscine administration 30 minutes prior to embryo transfer can significantly increase pregnancy rates by reducing uterine cramps (13).

Piroxicam is another NSAID which has been used before embryo transfer in various studies with different results. Its mechanism of action, although being similar to other NSAIDS, is not completely understood, but may be related to prevention of prostaglandin synthesis by a reversible inhibition of the cyclo-oxygenase enzyme (14).

Due to the controversies in different surveys, this study was performed to assess the effects of administrating Piroxicam prior to embryo transfer in ART.

## Materials and Methods

This pilot study was performed in Vali-e-Asr Hospital from August 2010 through December 2011 after obtaining approval from the Ethical Committee of Tehran University of Medical Sciences in 230 patients who attended the infertility clinic. Inclusion criteria consisted of patients with the age group of 20-35 years old and with ART indication due to tubal factors, ovulation disorders or severe male factor. A long gonadotropin-releasing hormone (GnRH) analogue protocol for pituitary desensitization and recombinant follicle stimulating hormone (rFSH; Gonal-F, Merck Serono,Switzerland) were used for controlled ovarian hyperstimulation. Oocyte retrieval was performed 36-38 hours after human chorionic gonadotropin (HCG) administration which was given when at least two 18 mm follicles were detected. After microinjection, embryo formation and getting a written informed consent, fifty cases who had a good response (> 4 oocytes) during the controlled ovarian hyperstimulation (COH) for ART and who had embryos for transfer, were randomly divided into two groups. Group A received Piroxicam (10 mg, Tolid Daru, Iran) orally half an hour before embryo transfer and group B did not use any form of medication which is the conventional method used (control group). Embryo transfer was done using the Wallace catheter without sonographic control. The patients were asked about their feeling of lower abdominal pain which was considered as having or lacking cramps. Both groups rested for 30 minutes after embryo transfer. Systemic diseases and endometriosis were exclusion criteria. Demographic data, infertility history, endometrial thickness, number of oocytes and embryos transferred, presence of cramps after embryo transfer and ART outcome were recorded in a questionnaire and registered by SPSS version 16. Success rates were compared using chi-square and analysis of variance (ANOVA).

## Results

The demographic characteristics and infertility history in both groups showed no significant difference (Table 1). Mean endometrial thickness was 9.55 ± 2.06 mm and 9.68 ± 2.07 mm in groups A and B, respectively (p=0.82).

After embryo transfer, uterine muscle cramps were reported by 4 women (16%) in group B and none in group A (p=0.03). Seventeen pregnancies (34%) occurred in the 50 patients included in the trial with a pregnancy rate of 32% (n=8) and 36% (n=9) in groups A and B, respectively (Table 2).

**Table 1:** Demographic characteristics of the two groups under study


Group Characteristics		Control(group B) N=25	Piroxicam(group A) N=25	Total N= 50	P value

**Age of woman in yearsmean±SD**		27.68 ± 4.58	28.649 ± 4.32	28.16 ± 4.45	NS^**^
**Age of man in years mean±SD**		32.86 ± 4.02	34.09 ± 4.09	33.48 ± 4.05	NS^**^
**Type of infertility No.(%)**	**Primary**	20(80)	19(67)	39(78)	NS^*^
	**Secondary**	5(20)	6(24)	11(22)	
**Duration of infertility (Y)**		6.18 ± 3.37	6.70 ± 3.94	6.44 ± 3.63	NS^**^
**Cause of infertility No.(%)**	**Male**	17(68)	18(72)	35(70)	NS^*^
	**Female**	4(16)	5(20)	9(18)	
	**Both**	4(16)	2(8)	6(12)	


NS; Non-significant,*; Chi-square test and **; ANOVA.

**Table 2:** Comparison of outcomes of the two groups under study


Group Outcome	Piroxicam	Control	P value

**Abdominal muscle cramps No.(%)**	0	4(16)	0.03
β-hCG positive No. (%)*	8(32)	9(36)	NS


NS; Nonsignificant and *; Chi-square test.

## Discussion

In ART cycles, different factors contribute to the success rates. Technical aspects of embryo transfer is one of the most important factors, besides the high quality embryos and uterine receptivity. Even with an atraumatic transfer, endometrial reaction can be induced and affect ART (2).

In recent years, attention has been paid to reduce or prevent uterine contractions or cramps using prostaglandin inhibitors or sedatives before transfer. Indomethacin and piroxicam are the two mostly cited prostaglandin inhibitors used before embryo transfer (12, 14). Regarding indomethacin, in a study by Bernabeu et al. (12), implantation rates did not show significant difference in oocyte recipients who had received indomethacin before transfer. In another study conducted by our group (2009) to compare the effect of indomethacin and hyoscine, we showed that indomethacin and hyoscine reduce uterine cramps (p=0.04) as compared to the control group. The results of the same study also showed that pregnancy rate is significantly higher in the hyoscine as compared to the indomethacin or control groups (p<0.04) (13). Hyoscine as an anti-muscarinic drug is supposed to reduce cervical spasm (15). Sirohiwal et al. (16) showed in a study that using hyoscine suppository can reduce cervical resistance during labor.

In different studies, Piroxicam is considered as a controversial topic. Moon et al. (17) studied the effect of administrating 10 mg beta-cyclodextrin piroxicam, one and two hours prior to embryo transfer. In this study, pregnancy rate was found to be higher (46.8%) than the control group (27.6%). Firouzabadi et al. (18) studied the effect of giving a single dose of piroxicam before embryo transfer on implantation and pregnancy rates in *in vitro* fertilization (IVF) cycles. The implantation and clinical pregnancy rates were significantly higher in the piroxicam treatment group compared with the control group. In contrast to the above mentioned studies, our results showed that although giving piroxicam before embryo transfer prevents uterine cramps, it has no significant effect on pregnancy rates in ART cycles. Similar to the present study, Asgharnia and Mehrafza (19) showed that piroxicam has no significant role in pregnancy rate. Also, Dal Prato et al. (14) showed that piroxicam administration before intracytoplasmic sperm injection has no additional effect on pregnancy outcome. Production of inflammatory cytokines is important for successful implantation. Prostaglandins promote decidualization of the endometrium and implantation by increasing vascular permeability.

In rodents and rabbits, implantation can be interrupted by injection of prostaglandin inhibitors (20, 21). Just before implantation occurs, there is an increase in endometrial vascular permeability which can be prevented by indomethacin. Also, an increase in prostaglandin levels in implantation sites has been shown during very early stage of implantation (22). The prostaglandins are supposed to be secreted either by the endomtrium or the embryo. It has been shown that blastocysts in human as well as sheep, cows, rabbits, and mice produce and secrete prostaglandins (23). Regarding the endometrium, although decidual synthesis of prostaglandins occurs, but its level is much lower as compared to proliferative and secretory endometrium which can be due to high progesterone levels during pregnancy. Nevertheless, an increase in prostaglandin E2 (PE2) synthesis at the implantation site is mainly due to the response of some signaling factors from blastocyst, such as the platelet-activating factor, and correlates with an increase in vascular permeability (22, 24). It is now well-accepted that decidua-derived PE2 is one of the major regulators of trophoblastic invasion, activating other signaling proteins.

Since prostaglandins play a key role in implantation, due to the present evidence, it seems reasonable to omit NSAIDs administration as a means of reducing uterine contractions in embryo transfer procedure until further evidence can prove their benefits. In spite of our findings, since this study was performed on a limited number of patients and since other numerous factors are involved in this process, we recommend that more precise studies be performed on a wider scale in order to obtain more accurate results.

## Conclusion

It seems that piroxicam administration 30 minutes prior to embryo transfer can not significantly increase pregnancy rates, but can prevent or reduce uterine cramps after the procedure.
